# Types of Nursing Intervention to Reduce Impact of Bullying and Aggression on Nurses in the Workplace

**DOI:** 10.3390/healthcare10081463

**Published:** 2022-08-04

**Authors:** Iyus Yosep, Rohman Hikmat, Ai Mardhiyah

**Affiliations:** 1 Department of Mental Health, Faculty of Nursing, Universitas Padjadjaran, Jawa Barat 45363, Indonesia; 2 Faculty of Nursing, Universitas Padjadjaran, Jawa Barat 45363, Indonesia; 3 Department of Pediatric Nursing, Faculty of Nursing, Universitas Padjadjaran, Jawa Barat 45363, Indonesia

**Keywords:** bullying, intervention, nurses, workplace

## Abstract

The bullying of nurses in the workplace hurts the individuals and the work environment. Bullying can cause mental health problems, reduces the quality of nursing services, and reduces patient safety. The purpose of this study was to describe types of nursing interventions to reduce impact of bullying on nurses in the workplace. This study used the scoping review method to examine literature from the CINAHL, PubMed, and ProQuest databases. The keywords used in English are “bullying OR cyberbullying” AND “nurse” AND “workplace OR work-place” AND “nursing care OR nursing intervention”. The inclusion criteria were full text, randomized control trial or quasi-experiment design, English language, population of nurses, and the publication period of the last 10 years (2013–2022). We found nine articles that discussed nursing interventions designed to reduce the impact of bullying on nurses in the workplace. The sample in the study was in the range of 26–97 respondents. Most of the articles in this review used the quasi-experiment method. The study showed that nursing interventions to heal had negative effects on the bullying on nurses. There are three types of interventions employed to reduce the impact of bullying and aggression on nurses in the workplace, namely training programs, cognitive rehearsal programs, and education programs.

## 1. Introduction

Bullying is an aggressive behavior to someone that is intentionally and repeatedly in the form of physical or verbal abuse that causes another person to feel uncomfortable [1]. Bullying in nurses is a problem that often occurs in the workplace and needs serious handling. Bullying behavior in nurses occurs in the workplace with the aim of offending, humiliating, and debilitating, which is harmful, repetitive, and persistent, and is directed at one or more individuals [2,3]. Based on a previous study, 75% incidents of bullying happen in the workplace [4]. The reported prevalence of bullying behavior among nurses in European countries is 21.0–30.2% [5]. The reported frequency suggests a high prevalence in America, namely between 48.0 and 82.0% [6,7,8], while the reported prevalence of bullying behavior among nurses in Australia is around 37.3–61.0% [9,10]. In Korea, reported prevalence of bullying behavior in nurses reached 15.8%, and the reported incidence of bullying behavior in nurses in Indonesia reached 51.2% [11].

The causes of bullying in nurses include individuals, the work environment, and work-related factors. Individual factors in nurses include self-centeredness and immaturity [12], and work environment and organizational culture factors include values, customs, work rules, and shared habits [11,13,14,15]. Factors that can cause bullying in nurses in the workplace are job demands, support, work control, seniority, educational differences, patient rights, and poor leadership [13,14,15]. The previous study reported on perpetrators of bullying incidents in nurses conducted by supervisors 40.7%, managers (22%), colleagues 43%, patients 71%, and patients’ families 47% [12]. Regarding the form of bullying behavior that occurs in nurses, based on the results of the study, verbal bullying accounts for 40.4%, and includes behaviors such as insulting one’s personality, being humiliated in front of others, being blamed for something that is not one’s responsibility, and being treated disrespectfully [16]; moreover, physical bullying accounts for 43.3%, and includes behaviors such as the pushing, hitting, kicking, and of slapping nurses [17]. Bullying behaviors have a negative impact for nurses.

Bullying has negative effects on nurses in the workplace [18], including increased work pressure, decreased focus, physically tired, having trouble sleeping, and feeling uncomfortable [19]. Regarding the impact of bullying, mental health disorders, such as depression, anxiety, and post-traumatic stress disorders, accounted for accounted for 29.9% of respondents; 8.7% reported physical health disorders such as palpitations, headaches, increased blood pressure, fatigue and insomnia; 81% reported lower work motivation; 1.27% reported an increase in absenteeism; and 3.24% reported overwork [20,21,22,23,24]. Bullying can decrease the quality of nursing services (2.11%) and patient safety (1.93%) [25,26,27].

Interventions were needed to reduce and prevent the bullying of nurses in the workplace, as it can have a physical and psychological impact [26] and can reduce the quality of nursing care [25,27,28,29]. Several studies have stated that interventions can be carried out to prevent bullying behavior in nurses, such as leadership support [2,20,23], educational interventions [28,29,30], cognitive exercises [31,32,33], and anti-bullying policies and regulations [32]. The results of previous studies indicate that education for all workers in hospitals can reduce the incidence of bullying in hospitals [16].

Nurses, as health workers who provide comprehensive nursing care, have a role in reducing and preventing bullying. However, nurses only focus on reducing the impact of bullying on patients. Thus, the interventions taken to prevent and reduce bullying on nurses are not carried out. Therefore, the authors intend to conduct a systematic scoping review to describe interventions that can be carried out to reduce the impact of bullying on nurses.

## 2. Materials and Methods

### 2.1. Study Design

**Figure 1:**
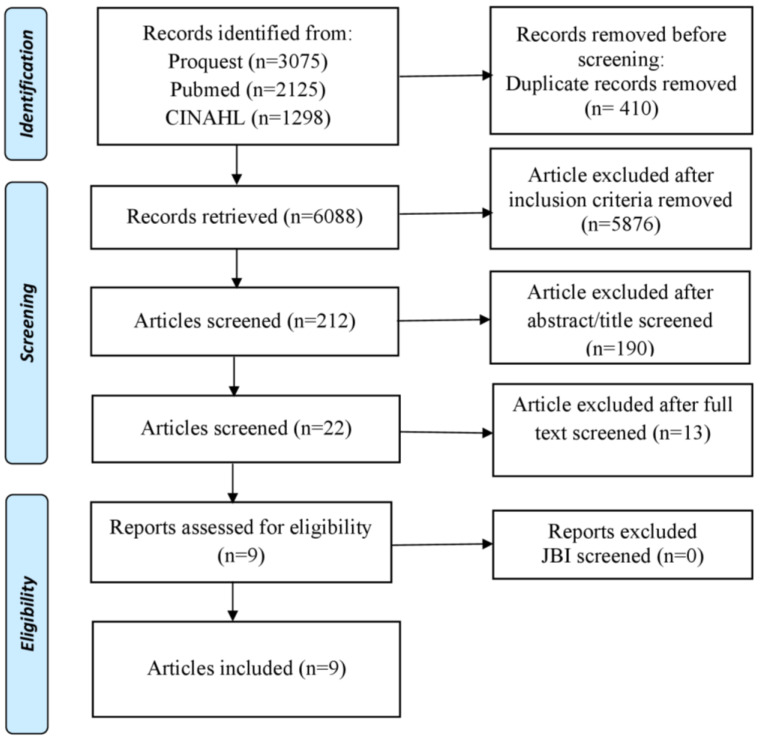
PRISMA Flow Diagram.

This study was designed as a systematic scoping review. We used 5 core stages, namely the identification of research questions, identification of relevant study results, study selection, data mapping, compilation of results, and reporting of study results [33]. This literature review used the PRISMA Extension for Scoping Reviews (PRISMA-ScR) to identify various topics that address interventions to reduce bullying behavior in nurses (Figure 1) [34].

### 2.2. Search Methods

The literature was collected from 3 databases, namely: CINAHL, PubMed, and ProQuest. The keywords used are: “bullying OR cyberbullying” AND “nurse” AND “workplace OR work-place” AND “nursing care OR nursing intervention”. The research question was: what are the interventions to reduce the impact and behavior of bullying on nurses in the workplace?

### 2.3. Inclusion and Exclusion Criteria

Articles were selected based on inclusion and exclusion criteria. The inclusion criteria of this study were: full text to be read by authors, randomized control trial or quasi-experiment design with nursing interventions, English language, that the population and samples were of nurses, and a publication period of the last 10 years (2013–2022) to analyze the latest research. The exclusion criteria of this study were studies that did not discuss interventions to reduce or prevent bullying or aggression in nurses in the workplace.

### 2.4. Data Extraction

The articles were manually extracted using tables by the authors. Before being included in the table, the author first analyzed and briefly summarized the contents of the reviewed study. In tabular form, it included the authors, year, country, study design, population and sample, procedures, interventions, and results of the study. Then, the authors described the results of the analysis, including an explanation of the interventions used to reduce the negative effects of bullying.

### 2.5. Quality Appraisal

Articles were analyzed using the Joanna Briggs Institute (JBI) critical assessment method. The assessment checklist based on The JBI Critical Appraisal contains several questions to assess the quality of the study [35]. The assessment criteria were given a score of ‘yes’, ‘no’, ‘unclear’, or ‘not applicable’, and each criterion with a score of ‘yes’ was given one point and another score was zero, and each study score was then calculated and added up. JBI Critical Appraisal tools have been approved by the JBI Scientific Committee following extensive peer review. The standard of a good article was determined by a score of above 75%, based on criteria and topic relevance.

### 2.6. Data Analysis

The articles collected were then read in full and analyzed by all authors. The author then discusses the interventions carried out in overcoming bullying against nurses. After discussing, the authors make a summary of each article reviewed. After being analyzed, the interventions from the literature were classified based on similar interventions and then described.

## 3. Results

The number of articles obtained from the search was 6498. After removing duplicate articles, we had 6088 articles. Furthermore, after elimination based on the inclusion criteria, there were 212 articles left. Then, after checking the title and abstract, 22 articles were found. Then, the author conducted an overall full-text analysis of the article and found nine articles to analyze.

The results showed articles from seven countries: there were two articles from Korea, two articles from Australia, and one article each from Turkey, Jordan, England, Iran, and the United States. The research designs used included five articles that used a quasi-experimental design and four articles that used a randomized control trial. Of the nine articles analyzed, there are three types of interventions that are effective in reducing the incidence and preventing bullying in nurses in the workplace: training program, cognitive rehearsal program, and education program.

Based on the results of the study, the training programs that can be carried out are assertiveness training, training programs, behavior management training, and workplace violence training programs. The training program focuses on training that involves all health workers in the workplace in preventing and reducing the incidence of bullying against nurses. Training is carried out over 4–8 weeks. The training is provided in the form of material delivery and demonstration. The cognitive rehearsal program includes several programs, namely the cognitive rehearsal program and the smartphone application for cognitive rehearsal intervention. Regarding cognitive rehearsal, the smartphone application-based program consists of introducing nonviolent conversation as standard communication, six web-toons on violent situations, and a bulletin board for questions and answers. The duration of the cognitive rehearsal program is 6–10 weeks. Moreover, the education program included education, a risk assessment checklist and prevention protocol, and clinical education. The education program is conducted from 2 days to 1 week. The form of the education about bullying provided is hybrid learning.

Articles were analyzed using the JBI Critical Appraisal Tool assessment method, with good article standards being denoted with scores above 75%, based on criteria and topic relevance (Table 1).

We found nine articles that discussed nursing intervention in reducing the impact and incidence of bullying on nurses in the workplace. We classified the interventions into three types, namely training programs, cognitive rehearsal programs, and education programs. The results of the analysis of the article are presented in tabular form as follows (Table 2):

## 4. Discussion

We found nine articles that discussed nursing interventions employed to reduce the impact and incidence of bullying and aggression towards nurses in the workplace. Bullying towards nurses included both physical and verbal behavior. Physical aggression included being pushed, hit, kicked, thrown, pinched, grabbed, and elbowed, while verbal bullying included labeling, yelling, and psychological bullying. Bullying causes someone to feel uncomfortable, pressured, afraid, and disappointed.

The study showed that bullying had physical, social, and psychological impacts on nurses in the workplace. The physical effects felt include difficulty sleeping, dizziness, and palpitations [44]. The perceived social impacts are apathy and anti-social. Moreover, the psychological impacts felt include lack of confidence, low self-esteem, shame, anger, helplessness, sadness, fear, negative thoughts, and traumatic experiences [4].

Nurses have an important role in reducing the impact and incidence of bullying on nurses in the workplace. Everyone needs to collaborate in improving the welfare of workers. Holistic nursing care to improve human health status, including victims of bullying, should be employed to reduce the impact and incidence of bullying.

Based on the study, of the types of nursing intervention employed to reduce the impact and incidence of bullying, there were training programs, cognitive rehearsal programs, and education programs. A previous study showed that a training program which was conducted for 8 weeks included empathy training and people management training [18]. There was aim to increase respect and reduce the bullying of nurses in the workplace [45]. Meanwhile, another study on cognitive rehearsal programs showed that interventions packaged with theatrics could reduce the incidence of bullying among nurses in hospitals [27]. In the education program intervention, education about the impact of bullying can reduce the incidence of bullying on nurses in the workplace because there is an understanding related to bullying [46].

Education, cognitive training, leadership style and organizational policies can help nurses to deal with bullying behavior [19,47,48,49]. In education and role-playing programs, nurses can recognize and deal with bullying behavior [24,31,50]. Moreover, a previous study used teaching courses provided by nurses with intensive skills units to overcome and manage bullying behavior [30], and another study mentioned that education can reduce the incidence of bullying. They provided nurses with information about bullying, communication, and conflict resolution in the form of workshops that could reduce verbal violence rates from 90% to 76%; then, nurses’ opinions were respected, and the work atmosphere improved [28]. Educational interventions are effective in reducing bullying behavior. Cognitive interventions could manage and identify bullying behavior in the workplace [49]. This requires policies that are supported by the leadership and bullying procedures to prevent bullying by an organization [48].

Interventions to reduce the impact of bullying on nurses need to be performed in collaboration with other professionals. This requires cooperation and coordination between various parties, especially health workers. Other professionals need to respect each other, increasing cooperation, and should not look down on the nursing profession. They should also work to improve policies to equalize work wages and ensure the quality of life of health workers in the workplace, especially nurses. Indeed, Intervention should not only be applied by and for nurses, as it requires collaboration and cooperation at the workplace level.

### Limitations

The limitations of this study were its small sample size taken from the literature. The article was limited to the last 10 years, therefore it does not discuss interventions carried out before then. The articles were only analyzed in English because of the author’s limitations in understanding other languages. The interventions reported were only carried out by nurses even though the cooperation between various parties in carrying out interventions to reduce the impact and incidence of bullying on nurses in the workplace is required. The discussion on how to reduce the impact of bullying and aggression is not comprehensive as the previous studies with the same theme are limited.

## 5. Conclusions

Nursing intervention can reduce the incidence and impact of bullying on nurses in the workplace. There are three types of nursing intervention, namely through a training program, cognitive rehearsal program, and an education program. Programs can be conducted online, hybrid, and offline for nurses and other employees in the workplace. These three types of interventions can be used as a basis for nurses in providing nursing care to prevent and reduce bullying in nurses in the workplace. In addition, nursing intervention can also improve the welfare and confidence of nurses. The recommendation in this study for further research was related to the effectiveness of nursing intervention to reduce the incidence and impact of bullying on nurses in the workplace.

## Figures and Tables

**Table 1:** JBI Critical Appraisal Tool.

Author, Published Year	JBI Critical Appraisal Tool	Study Design
[36]	88.9% (8/9)	Quasi-experimental
[37]	92.3% (12/13)	RCT
[38]	84.6% (11/13)	RCT
[39]	76.9% (10/13)	RCT
[40]	92.3% (12/13)	RCT
[41]	88.9% (8/9)	Quasi-experimental
[42]	77.8% (7/9)	Quasi-experimental
[30]	88.9% (8/9)	Quasi-experimental
[43]	100% (9//9)	Quasi-experimental

**Table 2:** Extracted data.

No	Author and Year	Purpose	Location	Method	Size Sample	Intervention	Result
1.	[36]	Effect of assertive training on the mobbing experienced on nurses	Turkey	Quasi-experimental	30	Assertiveness training	Decreased the impact of mobbing on nurses
2.	[37]	Effect of training programs on the impact of workplace violence on nurses in Jordan	Jordan	RCT	97	Training program	Decreased the incidence of violence and a decrease in the effect of violence on nurses.
3.	[38]	Effect of a cognitive exercise program (CRP) on the incidence of bullying in nurses	Korea	RCT	40	Cognitive rehearsal program	Significant effect on the incidence of bullying and improved interpersonal relationships in the work environment and increased adaptive coping.
4.	[39]	Effect of Cognitive Exercise Intervention (CRI) on the effect of bullying on nurses	Korea	RCT	72	Smartphone application for cognitive rehearsal intervention	Effective in reduced bullying on nurses and improved nurses’ well-being.
5.	[40]	Effect of behavior management training on the impact of aggressiveness on nurses	North Carolina, England	RCT	75	Behavior Management Training	Decreased the violent behavior towards nurses.
6.	[41]	Effect of educational programs on the prevention and incidence of violence against emergency nurses	Iran	Quasi-experimental	37	Education program, risk assessment checklist, and prevention protocol	Reduced the impact of violence and verbal abuse experienced by nurses.
7.	[42]	Effectiveness of clinical education in reducing the frequency of incidents of violence in nurses	Australia	Quasi-experimental	65	Clinical education	Effective in increasing knowledge about violence and reduced the incidence of verbal violence in nurses.
8.	[30]	Impact of cognitive exercise in reducing violent and negative actions against nurses	Boston (USA)	Quasi-experimental	26	Cognitive rehearsal	Effective in reducing rudeness and lateral violence in nurses and increased nurse resilience.
9.	[43]	Effect of workplace violence training program on the incidence of violence in nurses	Australia	Quasi-experimental	71	Workplace violence training program	Increased the confidence of nurses and reduced the negative effects of violence in nurses.

## Data Availability

Not applicable.
